# Socioeconomic position and health services use in Germany and Spain during the Great Recession

**DOI:** 10.1371/journal.pone.0183325

**Published:** 2017-08-30

**Authors:** Lourdes Lostao, Siegfried Geyer, Romana Albaladejo, Almudena Moreno-Lostao, Juana M. Santos, Enrique Regidor

**Affiliations:** 1 Department of Sociology, Medical Sociology, Universidad Pública de Navarra, Pamplona, Spain; 2 Medical Sociology Unit, Hannover Medical School, Hannover, Germany; 3 Department of Preventive Medicine and Public Health, Universidad Complutense de Madrid, Madrid, Spain; 4 Universidad de Navarra, Pamplona, Spain; 5 CIBER Epidemiología y Salud Pública (CIBERESP), Madrid, Spain; 6 Instituto de Investigación Sanitaria del Hospital Clínico San Carlos (IdISSC), Madrid, Spain; University of Groningen, NETHERLANDS

## Abstract

**Objective:**

The relationship of socioeconomic position with the use of health services may have changed with the emergence of the economic crisis. This study shows that relationship before and during the economic crisis, in Germany and in Spain.

**Methods:**

Data from the 2006 and 2011 Socio-Economic Panel carried out in Germany, and from the 2006 and 2011 National Health Surveys carried out in Spain were used. The health services investigated were physician consultations and hospitalization. The measures of socioeconomic position used were education and household income. The magnitude of the relationship between socioeconomic position and the use of each health services was estimated by calculating the percentage ratio by binary regression.

**Results:**

In Germany, in both periods, after adjusting for age, sex, type of health insurance and need for care, subjects belonging to the lower educational categories had a lower frequency of physician consultations, while those belonging to the lower income categories had a higher frequency of hospitalization. In the model comparing the two lower socioeconomic categories to the two higher categories, the percentage ratio for physician consultation by education was 0.97 (95%CI 0.96–0.98) in 2006 and 0.96 (95%CI 0.95–0.97) in 2011, and the percentage ratio for hospitalization by income was 1.14 (95%CI 1.05–1.25) in 2006 and 1.12 (95%CI 1.03–1.21) in 2011. In Spain, no significant socioeconomic differences were observed in either period in the frequency of use of these health services in the fully adjusted model.

**Conclusion:**

The results suggest that the economic crisis did not alter accessibility to the health system in either country, given that the socioeconomic pattern in the use of these health services was similar before and during the crisis in both countries.

## Introduction

In wealthy countries with universal healthcare coverage, several studies have observed that the probability of consulting a general practitioner and of hospitalization does not vary across income or socioeconomic groups, or is even more frequent in persons belonging to low socioeconomic groups [1–7]. However, this relationship may have changed after the emergence of the economic crisis in 2008.

A number of circumstances related to the economic crisis of 2008 may have led to lower use of health services, and this reduction might have had a greater effect in low socioeconomic groups. For example, some authors suggest that employed people may have less time to go to a medical appointment, whereas those who are unemployed prefer to use their time looking for a job rather than going to the doctor [8]. An added circumstance not mentioned by these authors is that restrictions in the assignment of resources to the healthcare sysem in many European countries may have brought about reduced access to the health system [9–10].

The lack of empirical evidence means we do not know whether the economic crisis has altered the principle of equity in health services use [11], one of the basic principles of health systems in advanced welfare countries. The variation in the economic situation in Europe offers the possibility to respond to this uncertainty by investigating what happened before and during the crisis in two countries where the magnitude and consequences of the crisis have been quite different: Germany and Spain. Whereas in Germany the unemployment rate dropped from 8.6% in 2007 to 5.0% in 2011, in Spain it rose from 8.4% in 2007 to 21.7% in 2011 [12]. On the other hand, the annual growth in public spending for health in Germany went from 4.4% in 2007 to 3.9% in 2010 and 3.3% in 2011, while in Spain it dropped from 8.0% in 2007 to -2.2% in 2010 and to -3.3% in 2011 [13]. A possible increase in socioeconomic inequalities in the use of health services in Spain, but not in Germany, would support the idea that reducing public expenditure on health care during the crisis may alter the principle of equity in the use of health services. The specific objective of this investigation was to show the relationship between socioeconomic position and health services use, before and during the economic crisis, in Germany and in Spain.

## Methods

### Data sources

The data for Germany was taken from the 2006 and 2011 Socio-Economic Panel (SOEP). The SOEP is a nationwide longitudinal survey project located at the German Institute for Economic Research, which employs a two-stage stratified sampling design. The regional units of the first sampling stage correspond largely to the electoral districts for the German parliament from which households were drawn. A random route sampling point (voting district) was used to select the households. Within each household, all adults aged 16 or over were selected. The first wave was carried out in 1984, and regular follow-ups are conducted to keep up with recent developments. Panel attrition is compensated for by sampling new subjects each year in order to obtain a sufficiently large number of cases and to avoid biases in the composition of respondents. A detailed account of the SOEP and its data structure can be found in Haisken- DeNew et al [14].

The Spanish data were taken from the 2006 and 2011 National Health Surveys carried out by the Ministry of Health and the National Statistics Institute. These are the last two national health surveys conducted in Spain on the date of this study. The sampling framework was made up of the Spanish non-institutionalized population aged 16 or over. The survey had a two-stage sample design. The first-stage units were the census sections, and the second-stage units were the households in each of the selected sections. The households were selected by simple random selection, and one adult aged 16 or over was selected within each household. In both the German and Spanish surveys, information was collected by face-to-face interviews. For the present study we selected subjects under 75 years of age, since the probability of being institutionalized increases after that age. A detailed account of the National Health Surveys and its data structure can be found in the websites of Ministry of Health and the National Statistics Institute [15–16]

### Study variables

The health services investigated were physician consultations and hospitalization in each country. In the SOEP those interviewed were asked if they had visited a physician in the last 3 months, and those who confirmed it were asked about the number of consultations made. People were considered to have consulted a physician if they had made any type of consultation in the previous 3 months. In the Spanish National Health Survey, respondents were interviewed about the frequency of their physician visits, and had to choose between one of the following four alternatives: less than 4 weeks ago, between 4 weeks and one year, more than a year ago, and never. People were considered to have consulted a physician if they had made any type of consultation in the last year (the first two alternatives). In both the German and Spanish surveys, respondents were asked if they had been hospitalized overnight during the previous year. Those who answered in the affirmative were considered to have been hospitalized.

The measures of socioeconomic position used were education and household income. The categories included in each variable are shown in the Table 1. Education refers to the highest level of education completed by the respondent. In the German survey several classifications of education were used. In the present study we used the Comparative Analysis of Social Mobility in Industrial Nations (CASMIN) classification of education. Based on this classification, subjects were grouped into the following four categories: tertiary education (codes 3a and 3b in CASMIN), full secondary education (codes 2c_general and 2c_vocational), intermediate secondary education (2b and 2a) and elementary education (codes 1a, 1b and 1c). In the Spanish survey we used the 1997 International Standard Classification of Education (1997 ISCED), and subjects were grouped into the following four categories: tertiary education (codes 5 and 6 of ISCED), upper secondary education (codes 3 and 4), lower secondary education (code 2) and elementary education (codes 0 and 1).

**Table 1:** Categories included in each indicator of socioeconomic position.

Germany	Spain
**Education**	
Comparative Analysis of Social Mobility in Industrial Nations (CASMIN)*	1997 International Standard Classification of Education (1997 ISCED)*
Tertiary education	Tertiary education
Full secondary education	Upper secondary education
Intermediate secondary education	Lower secondary education
Elementary education	Elementary education
**Income**	
Income based on open question in each year, and weighted by number of household members	Income based on closed question with various intervals (intervals in 2006 were different from those in 2011)
**2006**	**2006**
More than 3660 euros (high)	More than 1800 euros (high)
3660 to 2116 euros (medium-high)	1800 to 1201 euros (medium-high)
2115 to 1700 euros (medium-low)	1200 to 900 euros (medium-low)
Less than 1700 euros (low)	Less than 900 euros (low)
**2011**	**2011**
More than 3998 euros (high)	More than 1850 euros (high)
3998 to 2652 euros (medium-high)	1850 to 1301 euros (medium-high)
2651 to 1800 euros (medium-low)	1300 to 800 euros (medium-low)
Less than 1800 euros (low)	Less than 800 euros (low)

*CASMIN and ISCED are two international classifications of education to which national education systems can be transferred. Both CASMIN and ISCED-97 basically distinguish between primary, secondary and tertiary education, and then differentiate further within these levels.

The SOEP database contains several measures of income based on the information on household income obtained from respondents. We used household income weighted by number of household members, in accordance with the recommendations of the OCDE [17]. For the statistical analysis, subjects were grouped into four categories using the quartile distribution in each year of study. About 1% of those interviewed did not answer the question about income. In the Spanish National Health Survey, household income was not obtained with an open question; rather, respondents were asked to select an income category from among several intervals shown in the questionnaire. For the statistical analysis, subjects were grouped into four categories. In the Spanish survey, the percentage of non response to the question on income was 11% in 2006 and 25% in 2011.

Self-rated health was used as the measure of general health that might increase the likelihood of health care utilization. In the German survey, self-perceived health was measured by the following question: “How would you describe your current health?” Respondents had to rate their health status by means of a five-point rating scale labelled by: very good, good, satisfactory, poor or bad. In the Spanish health survey, self-perceived health was measured by the following question: “Over the last 12 months, would you say your health has on the whole been very good, good, fair, poor or very poor?” Respondents were asked to choose one of the five alternatives in this survey.

Sex, age and type of health coverage were used in the analyses as confounding variables. In both the German and Spanish surveys, respondents were asked what type of health coverage they had. The responses were grouped into two categories: those who had only some type of public healthcare coverage, and all others. All others included both those individuals who had only private insurance and those who had both public and private health insurance.

### Statistical analysis

For each country we estimated the frequency–as a percentage–of respondents who had consulted a physician as well as the percentage of respondents who had had any hospital admission according to the two measures of socioeconomic position. We then estimated the magnitude of the relationship between socioeconomic position and the use of each health service by calculating the percentage ratio estimated by binary regression, by using subjects included in the highest socioeconomic category as the reference group. The variables included in the regression models as possible confounders and/or as indicators of the need for care were age, sex, self-rated health and health coverage. We have estimated two models. In a first model, we have only included as adjustment variables age and sex. And in a second model, we have added self-rated health and health coverage, in order to evaluate if the magnitude of the association obtained in the first model is modified with the inclusion of the variables indicative of the need for care. Since income was collected as intervals in the Spanish surveys, it was not possible to construct a measure of income weighted by number of household members. However, in the analyses of the data from the Spanish survey we also included household size as a possible confounder when the measure of socioeconomic position was household income.

## Results

Table 2 shows the distribution of the population and the frequency of physician consultation and hospitalization in accordance with the two measures of socioeconomic position in Germany and Spain. With some exceptions, in most of the socioeconomic categories, the percentage of persons who consulted a physician or who had been hospitalized between 2006 and 2011 increased in Germany and decreased in Spain.

**Table 2:** Sample size, frequency (in percentage) of physician consultations and of hospitalization, by educational level of head of household and by household income. Germany and Spain, 2006 and 2011.

Country and indicator of socieconomic position ** **	Sample size (n)		Physician consultation^1^ (%)		Hospital admission^2^ (%)	
	2006	2011	2006	2011	2006	2011
**Germany**						
**Educational level**						
Tertiary	4,215	4,209	67.0	72.5	9.7	11.1
Full secondary	2,162	2,119	64.7	66.3	9.6	9.6
Intermediate secondary	5,857	5,533	64.5	69.0	9.9	12.3
Elementary	7,688	6,481	69.0	71.8	12.1	14.0
**Income**						
High	5,127	4955	64.9	68.0	8.3	9.8
Medium-high	5,114	4702	64.8	69.2	9.7	11.4
Medium-low	5,055	4624	68.8	71.4	12.0	13.6
Low	4,382	3933	69.1	73.8	12.6	15.3
**Spain**						
**Educational level**						
Tertiary	6,057	4,239	79.5	77.8	8.0	7.2
Upper secondary	5,258	4,018	80.9	79.5	8.0	7.3
Lower secondary	3,270	6,016	81.1	79.9	8.8	7.3
Elementary	10,865	3,446	84.3	83.3	10.1	9.4
**Income**						
High	6,542	4,190	80.7	79.0	7.1	7.4
Medium-high	6,175	2,689	81.2	79.9	8.9	6.9
Medium-low	4,788	3,849	81.9	80.6	8.8	7.6
Low	5,244	2,450	85.0	82.3	10.5	9.5

1. Physician consultation refers to the last 3 months in Germany and to the last year in Spain.

2. Hospital admission refers to the last year.

The relationship between the measures of socioeconomic position and physician consultation in 2006 and 2011 is presented in Table 3. In general, the percentage ratio adjusted for age and sex in the different socioeconomic categories did not differ significantly from the reference category in either country. After adjusting for age, sex, self-rated health and type of health insurance, the percentage ratio in Germany in each socioeconomic category of education and income was not statistically different from the reference category except in subjects with intermediate secondary education and elementary education, for whom it was 0.97 [95% confidence interval (95%CI) 0.95–0.99] and 0.96 (95%CI 0.95–0.98) in 2006 and 0.96 (95%CI 0.94–0.98) and 0.95 (95%CI 0.93–0.97) in 2011, respectively. In Spain, the percentage ratios in the fully adjusted model were not significantly different from the reference category.

**Table 3:** Physician consultation by educational level and income. Percentage ratio (PR) and 95% confidence interval (95% CI).

Country and indicator of socioeconomic position	2006								2011							
	Model 1				Model 2				Model 1				Model 2			
	PR	95% CI			PR	95% CI			PR	95% CI			PR	95% CI		
**Germany**																
**Educational level**																
Tertiary	1.00				1.00				1.00				1.00			
Full secondary	1.02	0.99	-	1.06	1.00	0.97	-	1.02	0.99	0.95	-	1.02	0.97	0.95	-	1.00
Intermediate secondary	0.99	0.96	-	1.02	0.97	0.95	-	0.99	0.97	0.95	-	1.00	0.96	0.94	-	0.98
Elementary	1.00	0.97	-	1.02	0.96	0.95	-	0.98	0.97	0.95	-	0.99	0.95	0.93	-	0.97
**Income**																
High	1.00				1.00				1.00				1.00			
Medium-high	1.01	0.98	-	1.03	1.00	0.98	-	1.03	1.02	0.99	-	1.05	0.99	0.96	-	1.01
Medium-low	1.03	1.01	-	1.06	1.01	0.98	-	1.03	1.01	0.98	-	1.03	0.99	0.97	-	1.01
Low	1.01	0.99	-	1.04	0.98	0.96	-	1.00	1.03	1.00	-	1.05	1.00	0.98	-	1.02
**Spain**																
**Educational level**																
Tertiary	1.00				1.00				1.00				1.00			
Upper secondary	1.01	1.00	-	1.03	1.01	0.99	-	1.02	1.03	1.01	-	1.05	1.00	0.99	-	1.02
Lower secondary	1.02	1.00	-	1.04	1.00	0.98	-	1.01	1.01	0.99	-	1.03	0.99	0.97	-	1.01
Elementary	1.00	0.99	-	1.01	0.99	0.98	-	1.00	1.01	0.99	-	1.03	0.98	0.96	-	1.00
**Income**																
High	1.00				1.00				1.00				1.00			
Medium-high	1.00	0.99	-	1.01	1.00	0.98	-	1.01	1.00	0.98	-	1.03	0.99	0.93	-	1.06
Medium-low	0.99	0.98	-	1.01	0.99	0.98	-	1.00	1.01	0.99	-	1.03	0.99	0.92	-	1.06
Low	0.99	0.98	-	1.01	0.99	0.97	-	1.00	1.02	1.00	-	1.04	0.98	0.90	-	1.06

Model 1. Adjusted for age and sex.

Model 2. Adjusted for age, sex, self-perceived health and type of health coverage.

The relationship between the measures of socioeconomic position and hospitalization are shown in Table 4. In Germany, the age- and sex-adjusted percentage ratio in 2006 was significantly higher in the two lower socioeconomic categories by income, and in the lowest category by educational level. After full adjustment, the percentage ratio decreased and did not show significant differences with respect to the reference category, except in subjects in the categories of medium-low and low income in 2006, for whom the percentage ratio was 1.23 (95%CI 1.08–1.39) and 1.22 (95%CI 1.07–1,39), respectively, and the category of low income in 2011, where it was 1.19 (95%CI 1.05–1.35). In Spain, the percentage ratio adjusted for age and sex was significantly higher in the two lower socioeconomic categories of education and income in 2006 and in the lowest socioeconomic category of education and income in 2011. After adjusting for age, sex, self-rated health and type of health insurance, the percentage ratio in all socioeconomic categories decreased and was not significantly different with respect to the reference category, except in the category of medium-high income, where it was 0.82 (95%CI 0.69–0.96).

**Table 4:** Hospitalization in the last year by educational level and income. Percentage ratio (PR) and 95% confidence interval (95% CI).

Country and indicator of socioeconomic position	2006								2011							
	Model 1				Model 2				Model 1				Model 2			
	PR	95% CI			PR	95% CI			PR	95% CI			PR	95% CI		
**Germany**																
**Educational level**																
Tertiary	1.00				1.00				1.00				1.00			
Full secondary	1.17	0.99	-	1.38	1.11	0.94	-	1.30	1.02	0.87	-	1.20	0.97	0.82	-	1.13
Intermediate secondary	1.11	0.99	-	1.26	1.05	0.92	-	1.18	1.18	1.06	-	1.32	1.07	0.95	-	1.20
Elementary	1.16	1.03	-	1.31	1.03	0.92	-	1.16	1.19	1.07	-	1.32	1.01	0.91	-	1.13
**Income**																
High	1.00				1.00				1.00				1.00			
Medium-high	1.17	1.04	-	1.33	1.12	0.99	-	1.27	1.15	1.03	-	1.29	1.07	0.95	-	1.20
Medium-low	1.33	1.18	-	1.50	1.23	1.08	-	1.39	1.25	1.11	-	1.39	1.10	0.98	-	1.24
Low	1.34	1.18	-	1.51	1.22	1.07	-	1.39	1.38	1.23	-	1.54	1.16	1.03	-	1.31
**Spain**																
**Educational level**																
Tertiary	1.00				1.00				1.00				1.00			
Upper secondary	1.09	0.97	-	1.22	1.01	0.90	-	1.13	1.06	0.92	-	1.22	0.98	0.84	-	1.13
Lower secondary	1.21	1.06	-	1.38	1.04	0.91	-	1.19	1.06	0.92	-	1.22	0.90	0.78	-	1.04
Elementary	1.12	1.01	-	1.25	0.93	0.84	-	1.04	1.25	1.06	-	1.47	0.95	0.80	-	1.12
**Income**																
High	1.00				1.00				1.00				1.00			
Medium-high	1.21	1.08	-	1.36	1.14	1.02	-	1.27	0.90	0.76	-	1.06	0.82	0.69	-	0.96
Medium-low	1.15	1.02	-	1.30	1.07	0.95	-	1.22	0.99	0.85	-	1.15	0.90	0.77	-	1.05
Low	1.22	1.08	-	1.39	1.11	0.98	-	1.27	1.22	1.03	-	1.44	1.00	0.84	-	1.20

Model 1. Adjusted for age and sex.

Model 2. Adjusted for age, sex, self-perceived health and type of health coverage.

## Discussion

### Main findings

Between 2006 and 2011, the frequency of physician consultation and of hospitalization increased in Germany and decreased in Spain. The two countries differed as to the pattern of physician consultation by education and of hospitalization by income. However, the socioeconomic pattern in the use of health services in each country was similar in the two periods analysed. In Germany, in both periods, after adjusting for age, sex, type of health insurance and proxy measure of the need for care (self-perceived health), subjects belonging to the lower educational categories had a lower frequency of physician consultations, while those belonging to the lower income categories had a higher frequency of hospitalization. In Spain, no significant socioeconomic differences were observed in either period in the frequency of use of these health services in the fully adjusted model.

### Comparison with other studies and possible explanations

The different evolution in the frequency of use of health services in Germany and Spain cannot be attributed to a different trend in the offer of health resources between 2006 and 2011, given that the trend was similar in both countries. Between 2006 and 2011 the number of physicians increased (from 3.5 to 3.8 per 1,000 inhabitants in Germany, and from 3.6 to 3.9 per 1,000 inhabitants in Spain), while the number of hospital beds decreased in both countries (from 8.3 to 8.2 per 1,000 inhabitants in Germany, and from 3.3 to 3.1 per 1,000 inhabitants in Spain) [18–19]. Nor can these trends be attributed to the emergence of some factor in either of these countries as a consequence of the economic crisis, given that, since the beginning of the present century, the trend in the frequency of use of these health services has been upward in Germany and downward in Spain [20].

Some factors associated with the economic crisis, such as the reduction in public expenditure dedicated to the health system, were much greater in Spain than in Germany [13]. The fact that the relationship between socioeconomic position and health services use in the fully adjusted model was similar in both countries in 2006 and 2011 suggests that this circumstance did not affect equity in the use of health services. In the case of Spain, the reduced expenditure was due basically to decreases in the salaries of health professionals as a result of measures taken by the Spanish government in 2010, and not to restrictions on the accessibility of health services. In fact, a study of health services use in European Union countries between 2007 and 2011 showed that Spain was one of the countries where citizens stated they had little or no difficulty in accessing physician consultations [21].

Several reasons may explain the different pattern of physician consultation by education in Germany and Spain. Previous investigations of health services use have shown that, in both countries, persons with lower educational level consult general practitioners more frequently than those with a higher level of education, whereas the opposite occurs in the case of consultations with specialist physicians [22–23]. In the present study, which analyses both types of consultations grouped together, the pattern of consultations with specialist physicians contributed in greater measure to the result in Germany than in Spain. The explanation for this can be found in the different way the health systems work. In the German system, patients can go directly to their chosen specialist physician, whereas in Spain the general practitioner is the gatekeeper to the health system, therefore the patient must be referred by a general practitioner before seeing a specialist [24]. As a result, the frequency of general practitioner and specialist consultations is very similar in Germany, whereas in Spain, general practitioner visits are three times more frequent than visits to specialists [22,25].

Another explanation that cannot be ruled out for the lower frequency of physician consultations in persons of lower educational level in Germany is the existence of health co-payments. Between 2004 and 2012, patients had to pay 10 euros per quarter for consultations with a general practitioner or specialist [24]. It is possible that persons with less education decide against such consultations, given that they generally also have a lower level of income. The consultation fee was introduced in order to reduce costs and to direct patients towards using general practitioners. This goal was however not accomplished. In general the consultation fee did not result in a reduction of health care utilization with the exception of an unintended side effect. According to a study and a government report patients with chronic diseases and those with the lowest incomes had reduced their health care utilization [26–28]. Apart from this notable exception patterns of outpatient visits are rather driven by patient preferences instead of the ability to pay a supplementary fee. Patients with lower educational level are preferring general practitioners over specialists while the opposite holds for patients with higher educational level.

No differences were seen in physician consultation by income in either Germany or Spain. Some international comparative studies in various countries have shown a higher frequency of physician consultations in persons with higher income in both countries [7,29]. These findings must be attributed to the higher frequency of specialist visits in persons with a higher level of income. However, some studies in Germany and Spain do not show the same pattern as reported in international studies. For example, in Germany, one study found a higher number of physician visits among persons with lower income [30], while another did not find a clear relationship between income and frequency of specialist visits [31]. In Spain, two previous investigations did not find a clear relationship between income and general practitioner or specialist visits, except in the case of those to private specialist [23,32].

Nor does it appear that co-payments for hospitalization in Germany (10 euros/day for hospital admissions up to a maximum of 28 days/year) [24] have greatly affected persons in low socioeconomic position. What is more, when subjects were classified by income, those with the lowest incomes had the highest frequency of hospitalization after adjusting for age, sex, type of health coverage and need for health care. A previous study also showed a greater number of hospitalizations in subjects with lower incomes [30]. The reasons for this finding are unknown. A previous investigation in Germany did not find any socioeconomic differences in the hospitalization of children and adolescents, except for the most severe health problems, in which those in lower socioeconomic position were found to have been hospitalized longer [33]. It may be that lower income persons avoid or delay physician consultation and that the higher frequency of hospitalization is therefore due to greater disease severity due to delay in seeking help.

In Spain, the same as in two previous studies [23,32], we did not find differences in hospitalization according to education or income. The only exception was in subjects with medium-high incomes who had a lower frequency of hospitalization in the second period. A previous study showed an important reduction in negative self-perceived health in the Spanish population during the economic crisis [34]. The present study confirms these findings (data not shown), as shown in the fact that the percentage of subjects with negative self-perceived health (fair, poor or very poor) was lower in 2011 (28.1%) than in 2006 (33.5%). However, among subjects with medium-high incomes, this decline was smaller. Perhaps this is why this socioeconomic group shows the lowest frequency of hospitalization, after adjusting for need for care.

### Strengths and limitations

One of the strengths of this study is that we compared the socioeconomic pattern in the use of health services in two countries where the economic crisis has had a different impact. Furthermore, we used the same data source in each country, before and during the economic crisis, therefore the variables related to the use of services and to socioeconomic position were the same. In the case of Spain, it should not rule out an education classification bias in 2006, since the number of subjects with elementary studies is too high and the number of subjects with lower secondary education is too low. In any case, the influence of this bias has been minimal, as in the fully adjusted model there were no significant differences in the use of health services in these two categories with respect to the reference category. Likewise, non-response to the question on income was much higher in the second period. Nevertheless, in a sensitivity analysis, we observed that the percentage of non response was similar in all categories of educational level (data not shown), which suggests that the impact on the results are likely to be low. On the other hand, a previous investigation in Germany using a socioeconomic indicator combining education, income and occupation found that subjects who were more socially disadvantaged less frequently consulted general practitioners than those who were more advantaged, whereas the opposite occurred in visits to the specialist physicians [35]. The present study, which analysed education and income separately, suggests that the results may differ depending on the measure of socioeconomic position used.

Finally, the limitation of considering self-perceived health as an indicator of the need for care should be taken into account. Some authors have stated that we cannot rule out an overestimation of health problems in persons of low socioeconomic position when they report negative self-perceived health [36]. In any case, such an overestimation does not necessarily differ over time, therefore it cannot affect the magnitude of the relationship between socioeconomic position and the use of health services. Also, it is unknown whether in low-income Germans self-rated health is a good indicator of the need for hospitalization, since the excess hospitalization in this population did not disappear after adjusting for that health measure.

### Conclusion

The results of the present study on the frequency of physician consultation and hospitalization in Germany and Spain by education and income suggest that the economic crisis did not alter accessibility to the health system in either country, given that the socioeconomic pattern in the use of these health services was similar before and during the crisis in both countries.
